# Withaferin A inhibits lymphocyte proliferation, dendritic cell maturation in vitro and prolongs islet allograft survival

**DOI:** 10.1038/s41598-021-90181-y

**Published:** 2021-05-21

**Authors:** Kenjiro Kumano, Mazhar A. Kanak, Prathab Balaji Saravanan, J. P. Blanck, Yang Liu, Srividya Vasu, Michael Lawrence, Bashoo Naziruddin

**Affiliations:** 1 Islet Cell Lab, Baylor University Medical Center, Dallas, TX USA; 2 Division of Transplant Surgery, Department of Surgery, Virginia Commonwealth University School of Medicine, Richmond, VA USA; 3 Flow Cytometry Core Laboratory, Baylor Scott and White Research Institute, Dallas, TX USA; 4 Baylor Simmons Transplant Institute, Baylor University Medical Center, 3500 Gaston Ave., Dallas, TX 75246 USA

**Keywords:** Immunology, Endocrinology, Medical research

## Abstract

The immunosuppressive regimen for clinical allogeneic islet transplantation uses beta cell–toxic compounds such as tacrolimus that cause islet graft loss. Previously we reported that the plant-derived steroidal lactone Withaferin A (WA) can protect islet grafts by inhibiting nuclear factor-kappa B (NF-κB). Since the NF-κB signaling pathway is essential for T-cell activation, we hypothesized that long-term WA administration may also provide an immunosuppressive effect. Treatment of BALB/c donor islets and C57BL/6N recipients with WA alone resulted in 80% islet graft long-term survival vs. 40% in low-dose FK506-treated mice. In vitro, WA significantly blocked mouse and human T-cell proliferation by CD3/CD28 bead stimulation and in mixed lymphocyte reaction assay. Treatment of immature dendritic cells with WA prevented their maturation in response to inflammatory stimuli, as seen by decreased expression of CD83 and human leukocyte antigen–DR isotype. Exosomes released by islets treated with WA contained significantly fewer proinflammatory molecules interleukin-6, interleukin-8, monocyte chemoattractant protein-1, interferon-gamma-induced protein-10, inducible nitric oxide synthase, and cyclooxygenase-2. In conclusion, WA treatment not only reduced inflammation but also prolonged allograft survival, possibly through suppression of dendritic cell maturation and T-cell proliferation. WA has the potential to inhibit both the innate and adaptive immune response to prolong allograft survival.

## Introduction

Clinical allogeneic islet transplantation is a promising procedure for the treatment of type 1 diabetes mellitus^1–3^. Current combination therapy with T-cell depletion and anti-tumor necrosis factor-α (TNF-α) antibody has enhanced graft survival^3^. However, functional decline and graft loss remain barriers for the long-term success of islet transplantation. Moreover, doses of systemic immunosuppressive drugs should be decreased to minimize islet toxicity and side effects such as infection and oncogenesis^4–6^.

Allorejection caused by T-cell-mediated immune reactions is one of the major problems that lead to islet graft loss. To date, no protocol has been able to control allorejection while preserving islet graft function^5^. Host CD8^+^ T cells recognize allogeneic major histocompatibility complex (MHC) class I peptide complexes as alloantigens through T-cell receptors (TCR), while host CD4^+^ T cells recognize allogeneic MHC class II similarly^7–10^. Mature dendritic cells (DCs) can sensitize alloreactive T cells via both direct and indirect antigen presentation^7^. The TCR signaling pathway for proliferation and differentiation of effector T cells requires the synchronized activation of activator protein 1 (AP-1), nuclear factor of activated T cells (NFAT), and nuclear factor-kappa B (NF-κB) transcription factors^11–14^. Blocking the activation of one of these transcription factors can significantly affect T lymphocyte activation and proliferation in response to allogeneic stimuli. Thus, blockade of the NF-κB pathway is an effective strategy to suppress the allogeneic response.

We have previously shown that the release of “isletokines” (cytokines and chemokines from islets) in response to inflammatory and metabolic stress contributes to graft dysfunction after islet transplantation in mice^15^. These isletokines are packaged into extracellular vesicles called exosomes, which are taken up by immune cells such as DCs, further exacerbating the allogeneic response^16,17^. Thus, reduction of islet stress and damage, in addition to inhibiting alloreaction, is important in preserving islet function and survival after transplantation.

In this regard, we investigated Withaferin A (WA), a natural steroidal lactone isolated from *Withania somnifera*, known for a broad range of medicinal properties including its antiinflammatory and immunomodulatory activities^18,19^. Previous reports including our own have shown that mechanistically, WA inhibits NF-κB activation by binding to inhibitor of NF-κB subunit beta (IKKβ), preventing phosphorylation of Iκβ^20–22^. NF-κB activation has an important role in chronic pancreatitis, and WA was able to block the progression of chronic pancreatitis in mice^23^. Our previous report showed that WA prevents Iκβ degradation and subsequent association of NF-κB with inducible nitric oxide synthase (iNOS) promoter in human islets, contributing to suppression of cytokine and chemokine release in vitro, and improved islet graft function in a syngeneic mouse transplant model^20^. We hypothesized that WA suppresses cytokine release, inflammatory response, and adaptive immune response in allogeneic islet transplantation. In this study, we investigated the immunosuppressive and immunomodulation abilities of WA and its effect on long-term islet allograft survival.

## Results

### WA treatment prolonged islet allograft survival in mice

**Figure 1:**
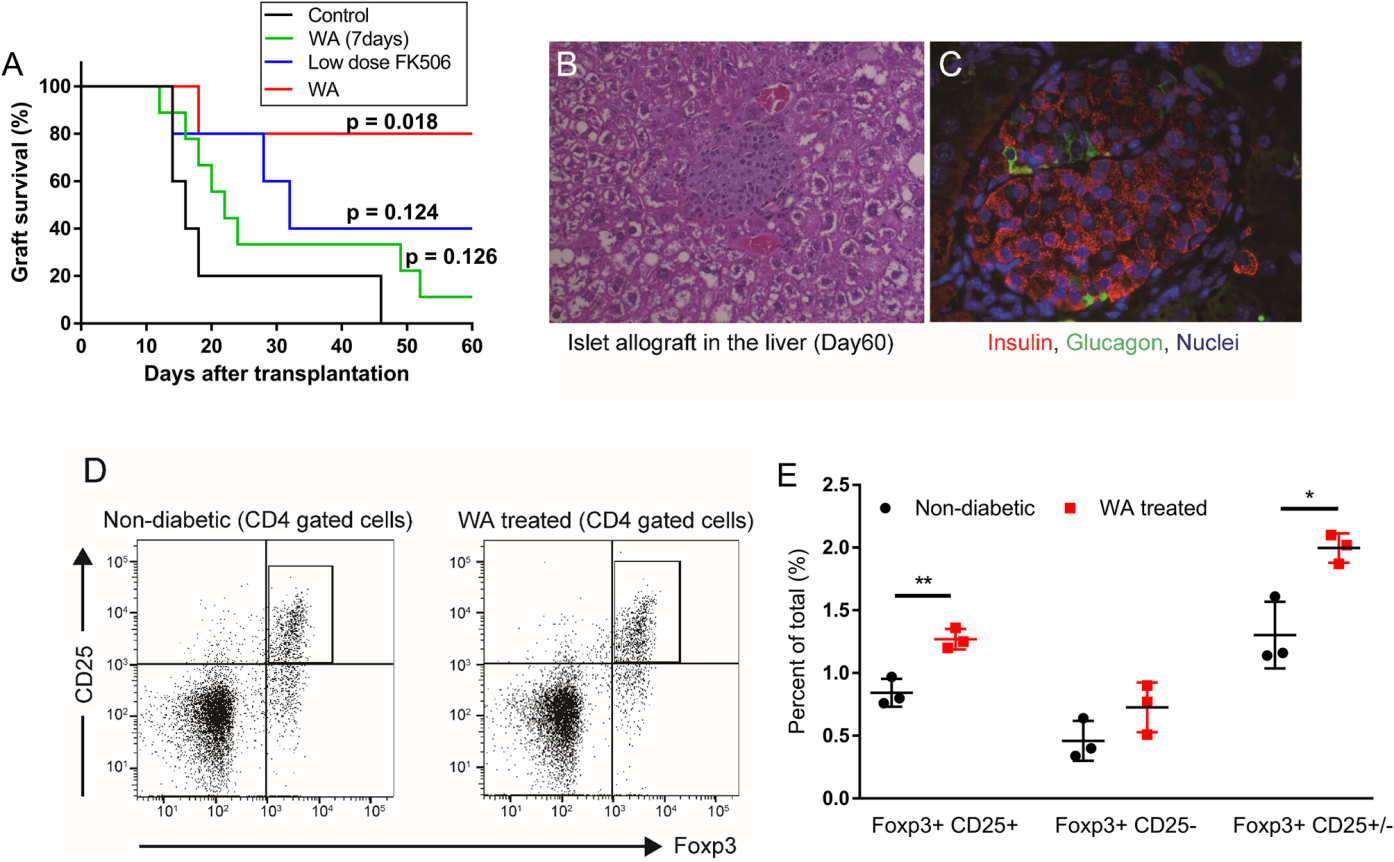
Effects of WA treatment on islet allograft survival. Donor BALB/c islets were isolated by collagenase containing 0.5 μg/mL WA. The islets were pretreated with WA 0.5 μg/mL for 30 min before islet transplantation, and then 300 islets were transplanted into the portal vein. (**A**) Kaplan–Meier plot for the survival of islet allografts (BALB/c to C57BL/6N) in recipient mice [n = 5 in control, low-dose FK506-treated (*P* = 0.124), and 1.25 mg/kg WA-treated groups; n = 5 in 1.25 mg/kg WA daily treatment group (*P* = 0.018), n = 9 in 1.25 mg/kg WA 7-day treatment group (*P* = 0.126)]. (**B**,**C**) Histological analysis of the liver grafts of the daily WA treatment group at autopsy, 60–70 days after transplantation (left panel, hematoxylin and eosin, × 100; right panel, anti-insulin and anti-glucagon antibodies, × 400). (**D**) Flow cytometry analysis of Treg population of the spleen in the WA daily treatment group. An autopsy study of the spleen was performed at the same time as liver autopsy (n = 3). CD4^+^ CD25^+^ Foxp3^+^ cells are gated and compared to those of nondiabetic control mice (n = 3). (**E**) Frequency of CD4^+^ CD25^+^ Foxp3^+^, CD4^+^ CD25^+^ Foxp3^−^ and CD4^+^ CD25^+/−^ Foxp3^+^ cell populations (Percent of total). *P < 0.05, **P < 0.01.

We initially investigated the effects of WA treatment on islet allograft survival in an acute rejection model by transplanting BALB/c islets into C57BL/6N mice. As shown in Fig. 1A, the WA treatment group showed the longest engraftment among the 4 groups (*P* = 0.018). The median survival times of the control (n = 5), WA 7-day treatment (n = 9), FK506 daily treatment (n = 5), and WA treatment (n = 5) groups were 16, 22, 32, and 60 days, respectively. There was a significant difference in graft survival between the WA treatment group and control (*P* = 0.018). On the other hand, WA 7-day treatment (*P* = 0.126) and low-dose FK506 (*P* = 0.124) treatment did not prolong graft survival. Over the period of 60 days, non-fasting blood glucose levels steadily increased in control (all 5), acute WA treated (8/9) and FK506 groups (3/5) from 10 to 13 days after transplantation. WA treatment group (4/5) remained normoglycemic over 60 days after transplantation (Supplementary Fig. S1). An autopsy of the liver and spleen was performed on the long-surviving recipient mice at 60–70 days after transplantation. Histological examination revealed intact islets engrafted in the liver of the WA treatment group (Fig. 1B,C). Moreover, flow cytometry analysis of the spleen revealed that the Treg (CD4^+^CD25^+^FoxP3^+^) population and CD4^+^CD25^+/−^FoxP3^+^ in the WA treatment group was significantly higher than that of nondiabetic control mice (1.27 ± 0.06% vs 0.84 ± 0.09%, *P* < 0.01; 1.3 ± 0.2% vs 2 ± 0.09%, *P* < 0.05) (Fig. 1D,E).

### WA prevents immune cell proliferation in mice

**Figure 2:**
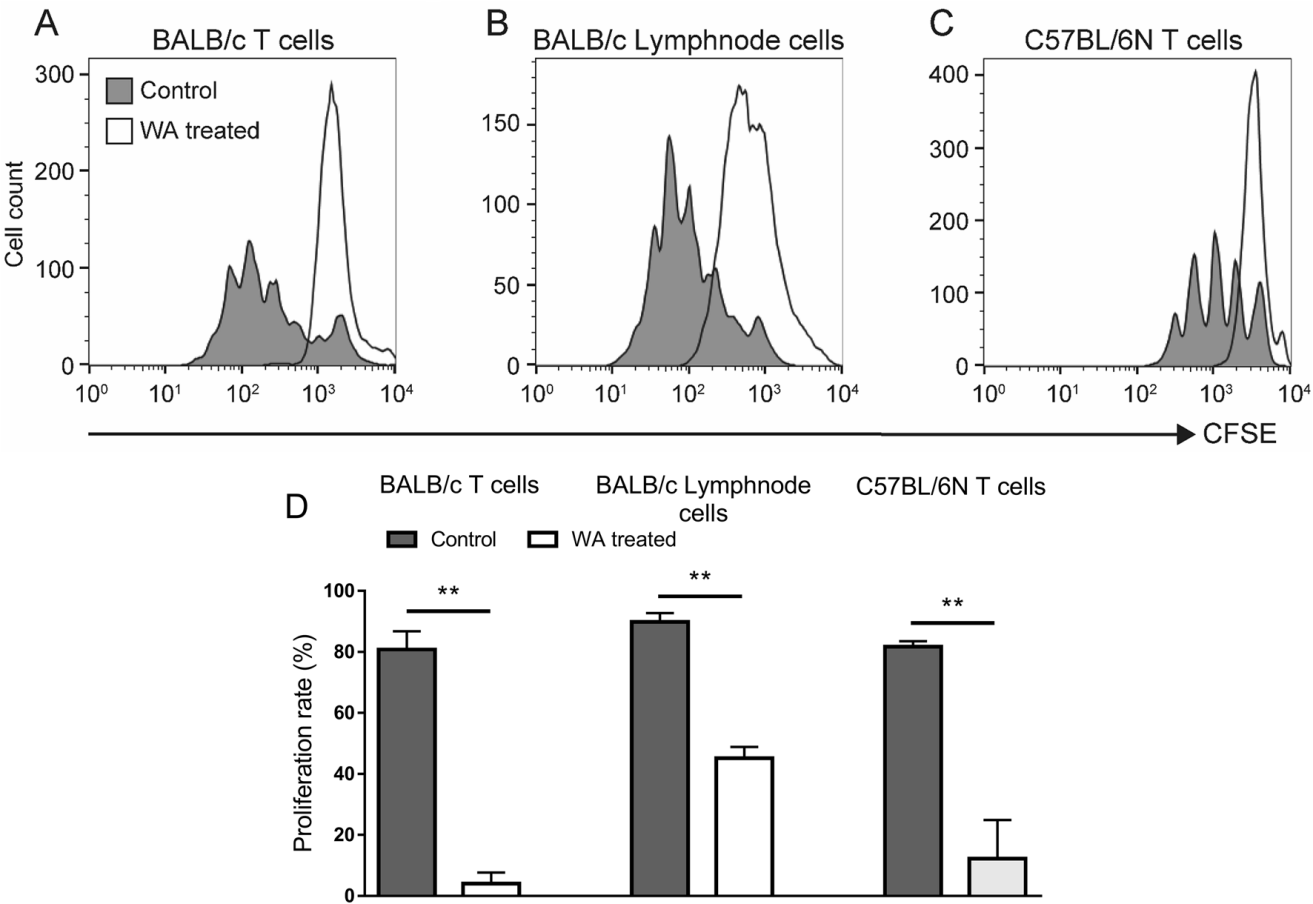
Effects of WA treatment on prevention of stimulated mouse lymphocyte proliferation. CD3^+^ T cell proliferation detected by flow cytometry analysis of CFSE-stained cells after 5 days of culture for (**A**) T cells from splenocytes isolated from BALB/c mice and stimulated by CD3/CD28 beads with or without WA 0.5 μg/mL treatment; (**B**) lymph node cells isolated from BALB/c mice and stimulated by CD3/CD28 beads with or without WA 0.5 μg/mL treatment; and (**C**) T cells from splenocytes isolated from C57BL/6N mice and stimulated by CD3/CD28 beads with or without WA 0.5 μg/mL treatment. Representative histograms from three independent experiments are shown. (**D**) Proliferation rate of BALB/c T cells, BALB/c lymphnode cells and C57BL/6N T cells are shown. **P < 0.01.

WA slightly reduced viability of T cells after 72 h exposure at concentrations of 0.5 µg/mL and 1.0 µg/mL (Viability (% of total): Control—66.8 ± 0.8%, WA 0.5 µg/mL—56 ± 4.5%, WA 1.0 µg/mL—55 ± 2.3%). Thus, we used concentrations of 0.25 and 0.5 µg/mL for further studies. We tested the effects of WA treatment on CD3/CD28 bead-induced proliferation of mouse T cells isolated from spleen and lymph nodes in vitro by fluorescence-activated cell sorting (FACS). As shown in Fig. 2A,B, splenocytes and lymph nodes treated with WA 0.5 μg/mL substantially inhibited the proliferation of BALB/c T cells compared with the control group after 5 days of culture. The proliferation of T cells derived from splenocytes of C57BL/6N was also suppressed compared with the control group (Fig. 2C). Proliferation rates are shown in Fig. 2D. These results indicate that WA treatment has an inhibitory effect on mouse lymphocyte activation and proliferation in vitro.

### WA suppresses human T-cell proliferation

**Figure 3:**
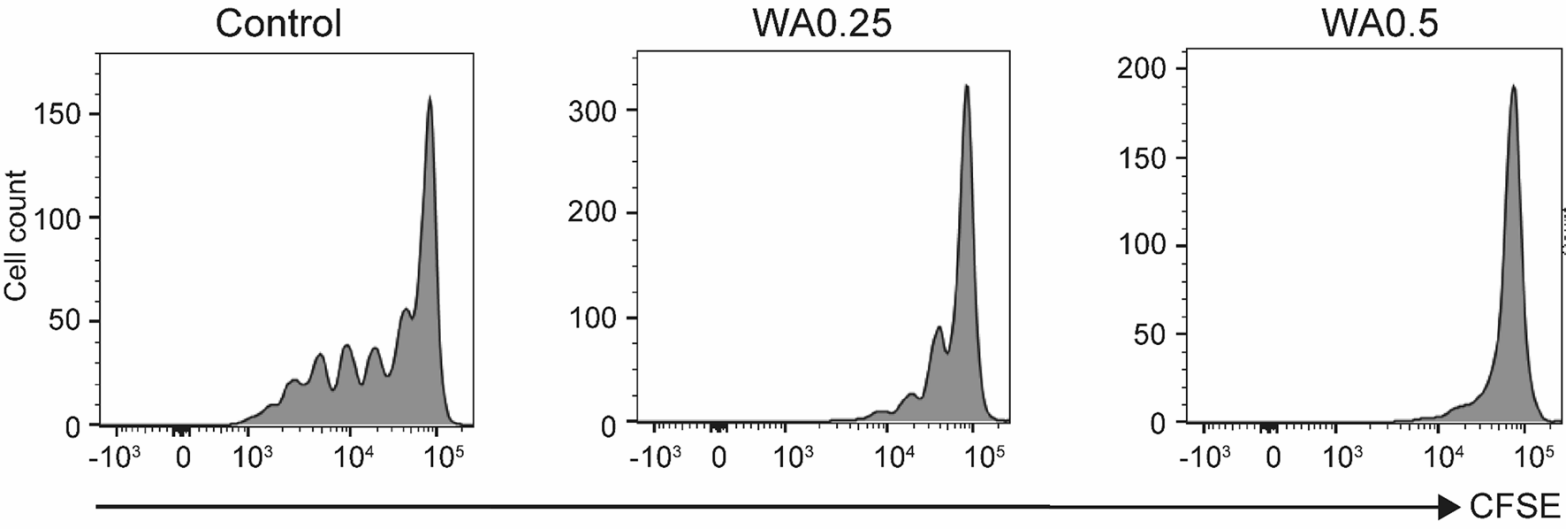
Effects of WA on human T-cell proliferation. Human PBMCs were isolated from the peripheral blood of healthy adult donors and stimulated by CD3/CD28 beads with or without WA 0.25 μg/mL (WA0.25) or WA 0.5 μg/mL (WA0.5) treatment. After 5 days of culture, CD4^+^ T-cell proliferation was detected by flow cytometry analysis of CFSE-stained cells. Representative histograms from three independent experiments are shown.

We assessed human PBMC-derived T-cell proliferation by FACS. Human PBMCs were stimulated by CD3/CD28 magnetic beads and proliferation was analyzed after 5 days of culture. The proliferation of human T cells was remarkably inhibited by WA in a dose-dependent manner (Fig. 3), suggesting that WA treatment suppresses both human and mouse T lymphocyte activation and proliferation in vitro.

### WA suppresses mixed human lymphocyte reaction

**Figure 4:**
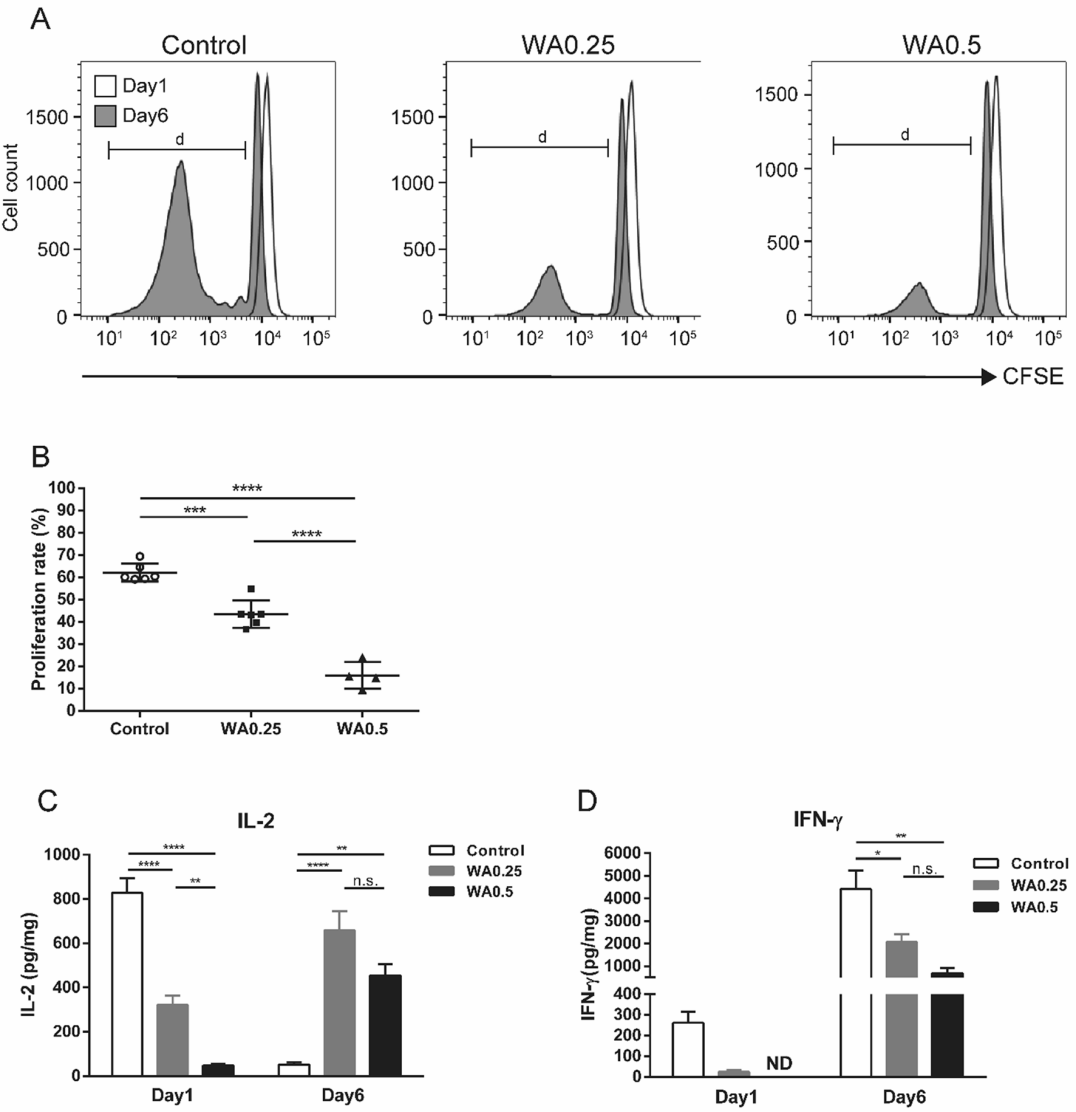
Suppression effect of WA on mixed human lymphocyte reactions. CFSE-labeled human PBMCs were used as responder cells. Mitomycin C-pretreated allogeneic human spleen cells were used as stimulator cells and were cocultured with or without WA 0.25 μg/mL (WA0.25) or WA 0.5 μg/mL (WA0.5) treatment. (**A**) Representative time-dependent change of CD4^+^ T-cell proliferation was detected by flow cytometry analysis of CFSE-stained cells. The clear histogram represents CD4^+^ T-cell proliferation at 1 day, while the gray area represents the cell proliferation at 6 days. (**B**) The proliferation rate (%) was calculated as a proportion of daughter cells at 6-day culture. (**C**,**D**) The levels of IL-2 or IFN-γ produced in the supernatants at day 1 and day 6 MLR were quantitated by enzyme-linked immunosorbent assay. Control (n = 6), WA0.25 (n = 6), WA0.5 (n = 4). *P < 0.05, **P < 0.01, ***P < 0.001, ****P < 0.0001. *ND* not detectable.

To confirm whether WA treatment suppresses an allogeneic response, we performed an MLR assay. Human PBMCs as responder cells were cocultured with allogeneic splenocytes as stimulator cells. The proliferation of human T cells derived from PBMCs was assessed after 6 days of culture. As shown in Fig. 4A, WA 0.25 μg/mL and 0.5 μg/mL treatment showed an inhibitory effect on the proliferation of CD4^+^ T cells. For quantitative analysis of T-cell proliferation, the proliferation rate was calculated as the percentage of T cells proliferating from the parent population, i.e., daughter population, after 6 days of culture (Fig. 4B). The proliferation rate of both WA 0.25 μg/mL (43.5 ± 2.5%) and 0.5 μg/mL (15.9 ± 3.0%) treatment was significantly lower than that of control (62.1 ± 1.7%) (*P* < 0.001, *P* < 0.0001, respectively) (Fig. 4B). Also, we measured the levels of interleukin (IL)-2 and interferon (IFN)-γ secreted in the culture supernatants at the day 1 and 6 MLR. As shown in Fig. 4C, there was a significant difference in the day 1 IL-2 level among the three groups (*P* < 0.0001), and WA 0.5 μg/mL treatment showed the lowest level (*P* < 0.0001 vs control, *P* < 0.01 vs WA 0.25). On the other hand, IL-2 levels of WA 0.25 and 0.5 μg/mL treatment at day 6 were higher than control (*P* < 0.0001, *P* < 0.01, respectively). IFN-γ levels of WA 0.5 μg/mL treatment at day 1 were below detection (< 4 pg/mL), and day 6 IFN-γ levels of WA 0.25 and 0.5 μg/mL treatment were significantly lower than control (*P* < 0.05, *P* < 0.01, respectively) (Fig. 4D). These results indicate that WA treatment has an inhibitory effect on the human allogeneic reaction in a dose-dependent manner. We also investigated whether WA treatment modulated the T helper cell phenotype during MLR, contributing to the induction of immune tolerance. We observed that WA decreased the population of CD8 + IFN-γ + T cells (*P* < 0.01) with an increase of CD4 + IL-4 + T cells (*P* = 0.079). However, the Treg population was not significantly changed compared to control (Supplementary Fig. S1).

### WA blocks the maturation of human monocytes into DCs

**Figure 5:**
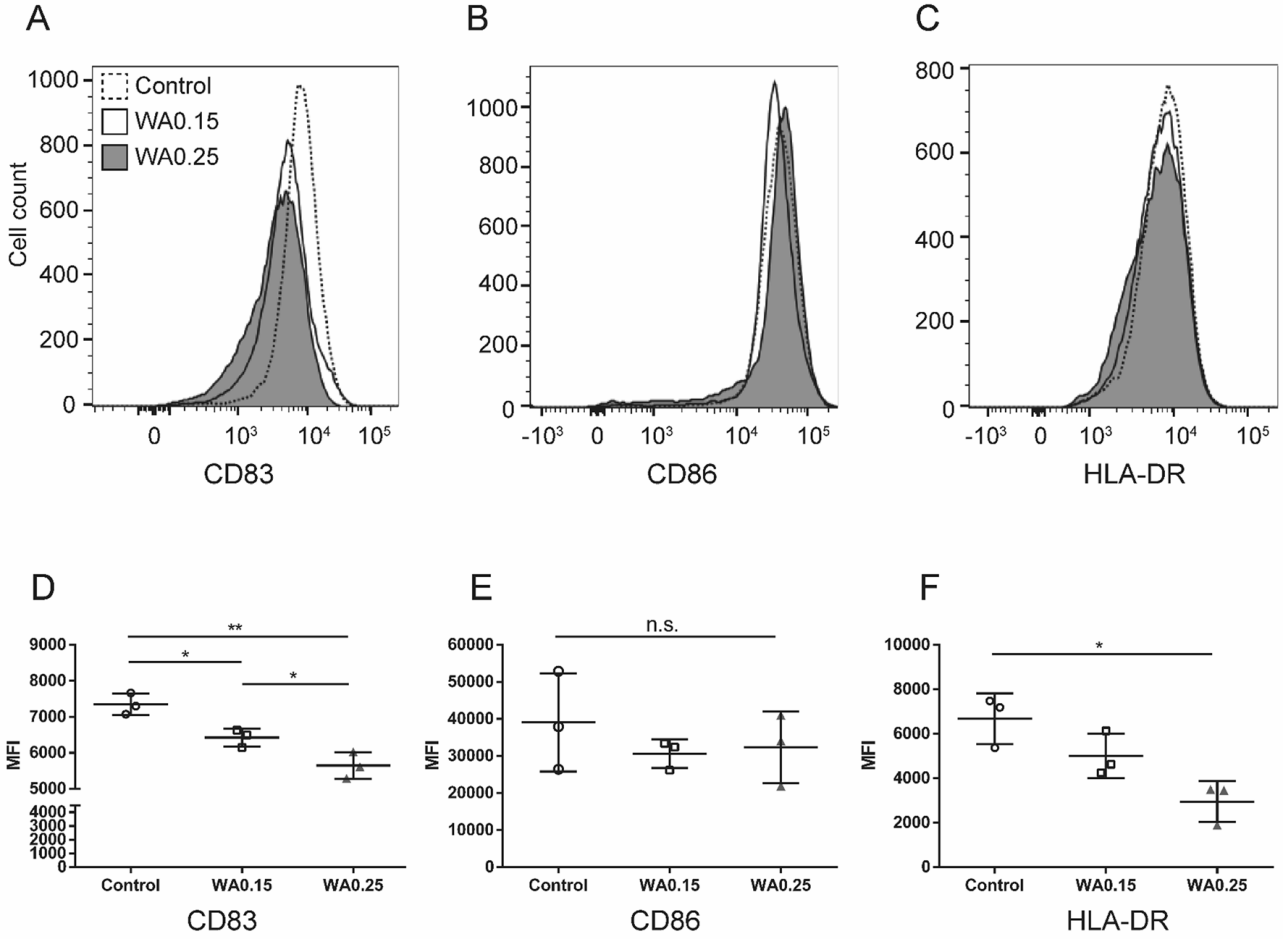
Inhibitory effect of WA treatment against human DC maturation. Human PBMCs isolated from healthy donors were cultured in a medium containing 0.15 or 0.25 μg/mL WA for the generation of mature DCs. Expression of (**A**) CD83, (**B**) CD86, and (**C**) HLA-DR was detected by flow cytometry after 48 h of culture. The dotted and clear histograms represent control cells and WA 0.15 μg/mL treated cells, respectively. The gray area represents WA 0.25 μg/mL–treated cells. (**D–F**) Median fluorescent intensity (MFI) of (**D**) CD83, (**E**) CD86, and (**F**) HLA-DR. Control (circle), WA0.15 (square), WA0.25 (gray triangle). *P < 0.05, **P < 0.01, n.s.; not significant. n = 3 independent experiments.

It is well known that mature DCs play a key role in the development of an allogeneic response^24^. We investigated whether WA treatment suppresses CD83 expression, resulting in the prevention of DC maturation. As shown in Fig. 5A, in contrast to mature DCs (control), DCs treated with WA (0.15 and 0.25 μg/mL) expressed low amounts of CD83. Quantitative analysis of CD83 using median fluorescent intensity showed that WA treatment (0.25 μg/mL) significantly suppressed CD83 expression (*P* < 0.05, Fig. 5D). On the other hand, there was no significant difference in CD86 expression between the control and WA treatment groups (Fig. 5B,E). Reduced HLA-DR expression in WA 0.15 and 0.25 μg/mL treatment in a dose-dependent manner was statistically significant compared to control (Fig. 5C,F). These results suggest that WA has an inhibitory effect on DC maturation by preventing CD83 and HLA-DR expression.

### WA suppresses exosomal isletokines released from cytokine-induced stressed human islets

**Figure 6:**
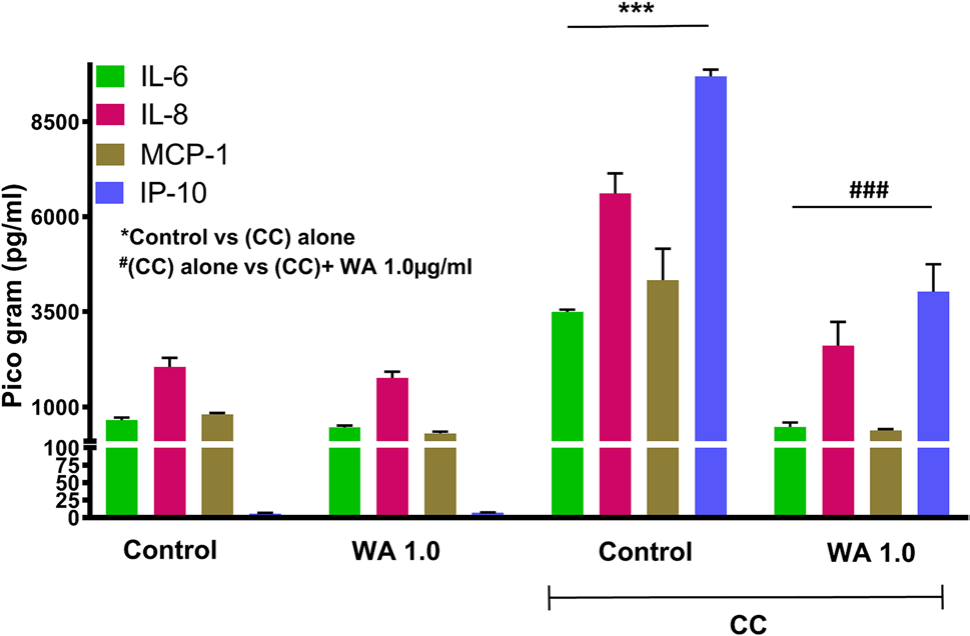
Ability of WA to control the release of isletokines via exosomes in cytokine-induced stressed human islets. Purified human islets of about 2500 islet equivalents (n = 3) were exposed to WA 1.0 μg/mL alone for 24 h or to a cytokine cocktail (CC) of IL-1β (100 U/mL) + IFN-γ (1000 U/mL) + TNF-α (1000 U/mL) for 24 h with or without 3 h of WA 1.0 μg/mL pretreatment. Untreated islets of approximately 2500 islet equivalents (n = 3), cultured for 24 h, were used as the control. The total amount of IL-6, IL-8, MCP-1, and IP-10 released in the culture via exosomes was quantified by ultrasensitive Luminex assay. The data show mean ± standard error of the mean. ***P < 0.001 vs control, ^###^*P* < 0.001 vs CC alone-treated islet group.

We previously showed that isletokines produced and secreted via exosomes in response to inflammatory and metabolic stress cause graft dysfunction after islet transplantation and that interferon-gamma-induced protein-10 (IP-10) expression of donor islets contributed to islet inflammation and loss of β cell function in mice^15^. We assessed whether WA prevents stressed islets from releasing isletokines in exosomes. Initially, exosomes were visualized using transmission electron microscopy and the size range of exosomes was confirmed to be within 50–200 nm vesicles that express exosome-specific markers CD9 and Flotillin-1 (Supplementary Fig. S2). As shown in Fig. 6, WA reduced levels of IL-6, IL-8, monocyte chemoattractant protein (MCP)-1, and IP-10 in exosomes released from islets exposed to cytokine cocktail when compared to control. Thus, WA protects islets from cytokine stress, leading to suppression of inflammatory exosomal isletokine release.

### WA suppresses human macrophage activation by reduction of exosomes released from stressed human islets

**Figure 7:**
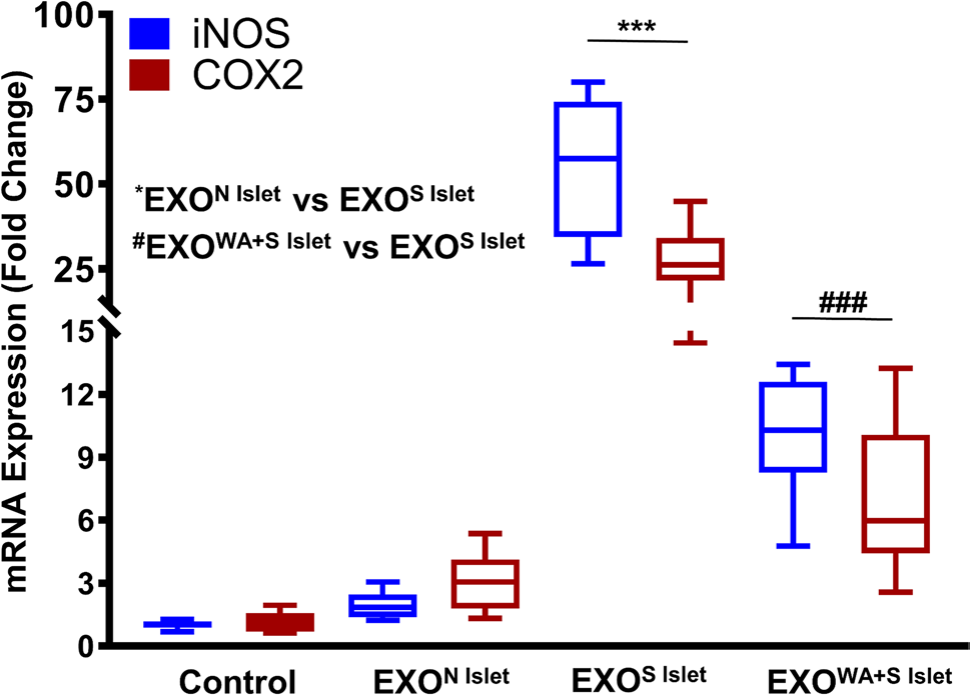
Protective effect of WA-pretreated human stress islet exosomes in the activation of human macrophages. Approximately 1 µg exosomes isolated from normal islets after 24 h of culture (EXO^N Islets^), exosomes isolated from cytokine cocktail (CC)-induced stressed islets (EXO^S Islet^), and exosomes isolated from WA 1.0 μg/mL-pretreated and CC-induced stressed islets (EXO^WA+S Islet^) were used for activation of human macrophages (THP cells), cultured for 6 h. The relative intracellular mRNA expression of iNOS and COX-2 was quantified by quantitative polymerase chain reaction. The data represent mean ± standard error of the mean. ***P < 0.001 compared to EXO^N Islets^, ^###^*P* < 0.001 compared with EXO^S Islets^.

Exosomes are known to stimulate antigen-presenting cells such as DCs and macrophages, and other lymphocytes by direct interaction. We investigated if exosomes released from control islets and stressed islets caused differential activation of the macrophage cell line THP-1 cells. Figure 7 shows that exosomes released from stressed islets cause elevation of inducible nitric oxide synthase (iNOS) and cyclooxygenase-2 (COX-2) mRNA expression in macrophages, indicating activation. On the other hand, mRNA levels of iNOS and COX-2 stimulated by exosomes released from WA-treated islets were significantly lower than those of nontreated islets. Thus, WA prevents exosomal isletokine-mediated activation of human macrophages.

## Discussion

WA has a long history of use in ancient Ayurvedic folk medicine as an anti-inflammatory drug. NF-κB plays a prominent role in inflammatory cytokine release and cell proliferation^25^, and several reports revealed the mechanism that WA inhibits NF-κB by preventing the formation of NF-κB essential modulator (NEMO)/IKKβ complex^21,22^. We previously demonstrated that WA treatment might apply to the field of transplantation, using a syngeneic islet transplant murine model^20^. WA has been shown to prevent an alloimmune cell reaction in vitro and graft-versus-host disease (GVHD) in a murine model^26^. However, there have been no reports on WA’s effect on allogeneic islet transplantation. In the present study, we demonstrated that WA inhibited mouse and human T-cell proliferation, prevented human DC maturation by reducing CD83 expression in vitro. WA treatment also prolonged graft survival of alloislet transplantation in mice.

In vivo, we treated not only recipients but also donor islets with WA before transplantation to maximize the effect of WA treatment. Transplanted islets are known to secrete damage-associated molecular patterns, including high-mobility group box 1 and isletokines in response to physiological stress, which facilitates an instant blood-mediated inflammatory reaction—an innate response leading to graft loss^27–29^. Moreover, we previously showed that cytokine-induced inflammation leads to induction of HLA class II expression on transplanted islet cells, potentially causing antidonor sensitization and adversely impacting allogeneic islet transplant outcomes^30^.

Donor cell treatment with WA before allogeneic transfer significantly prevented GVHD-associated mortality and decreased the levels of cytokines^26^. Our previous study also showed that WA did not affect viability or insulin secretory function but suppressed the release of inflammatory cytokines (MCP-1, IL-6, IL-8, IP-10) from isolated human islets in vitro^20^. The current study revealed that WA suppressed inflammatory cytokine release via exosomes in stressed human islets. It is known that IKKβ inhibitor (benzoxathiole) suppresses iNOS and COX-2 expression^31^. Indeed, several studies have shown that NF-κB directly binds to the iNOS and COX-2 promoter region and regulates transcription. Thus, we presumed that WA prevents activated macrophages, which have a central role in the innate immune response, from producing iNOS and COX-2 in inflammatory conditions^32,33^. As we expected, islets pretreated with WA produced less exosome in cytokine-induced stress conditions, which contributed to the suppression of macrophage activation. Given the contribution of islet derived factors including cytokines and chemokines including IP-10 to allograft response^34,35^, these findings suggest that donor islet treatment by WA might attenuate the alloresponse triggered by innate immune cells.

Regarding cytokine production in MLR, the IL-2 level on day 6 of WA treatment was significantly higher than that of control, despite small proliferation. Since it has been shown that IL-2 levels increase quickly following immune response and peak after several days^36^, the peak of IL-2 production might be delayed due to WA treatment.

Our in vivo results also showed that WA treatment has some limitations in the allogeneic response. First, WA treatment for 7 days after transplant did not extend allogenic graft survival compared to control, which means short-term WA administration during the post-transplant period is not sufficient. Second, although WA treatment achieved 80% graft survival, transcription factors such as NFAT and AP-1 also play a critical role in the TCR signaling pathway. For example, FK506 inhibits dephosphorylation of NFAT by blocking calcineurin activity, which dampens IL-2 production; thus, low-dose FK506 (0.5 mg/kg) administration resulted in 20% graft survival. Interestingly, it is reported that FK506 also inhibits NF-κB activation and suppresses inflammatory cytokine release^37^. Thus, combination therapy with low-dose FK506 and WA is considered an attractive synergic therapy to reduce the use of systemic immunosuppressants, which would contribute to the reduction of islet toxicity. Further investigations are necessary to determine whether combination therapy achieves superior transplant outcomes.

Of note, our study showed that WA prevents human DC maturation by reducing CD83 expression. CD83 used to be described as a highly specific marker for activated mature DCs in humans and mice, and the amino acid sequence is conserved well between the two^38^. Since NF-κB regulates the promoter of the CD83 gene^39–41^, we tested whether WA prevents CD83 expression on DCs during the process of maturation. Interestingly, our results showed that WA treatment decreased CD83 expression by approximately 20% compared with the nontreated mature DCs. On the other hand, CD86 expression was not suppressed. Thus, the suppressive effect of WA on CD83 expression is selective and mild.

Despite many studies on CD83 function, there are contradictory findings regarding its effect on DCs^38,42,43^. However, some reports show that a reduction of CD83 expression causes impaired mature DC function. Kruse et al. showed that inhibition of CD83 cell surface expression in mature DCs leads to a significant reduction of their T-cell stimulatory capacity in humans^44^. Moreover, Seldon et al. reported the efficacy of the human anti-CD83 monoclonal antibody on activated DCs in the human PBMC-SCID mouse xenograft model of GVHD^42^. The important role of CD83 in T-cell development became evident in CD83 knockout mice, resulting in a severe reduction of CD4^+^ T cells^45^. However, CD4^+^ T cells from CD83^−/−^ mice proliferated normally in response to TCR-induced proliferation (MLR). Thus, we did not examine whether WA reduces CD83 expression in T cells.

Importantly, our data showed that WA treatment slightly suppressed HLA-DR (human MHC class II) expression on DC, consistent with a previous report^26^, indicating its potential to inhibit MHC II mobilization and antigen presentation. We also found that the Treg population of splenocytes in the WA-treated group was significantly higher than in control nondiabetic mice. Oh et al. recently reported that immature DCs activate Treg development^46^. Moreover, partially mature DCs have been reported to have tolerogenic properties by induction of Tregs^47^. We confirmed that WA prevented complete maturation of immature DCs derived from human PBMCs by TNF-α stimulation, resulting in semimature DCs in vitro (data not shown). Since our in vitro study showed WA treatment does not induce Treg proliferation, the increase in the Treg population in vivo could be a result of tolerogenic DC-mediated Treg cell differentiation rather than proliferation, warranting further investigations. Taken together, our results suggest that blockade of DC maturation by WA may also contribute to the inhibitory effect on alloresponse and improvement of islet allograft transplant outcomes.

Although we have not focused on the molecular mechanisms of suppression of DCs and T cells in our current study, previous reports have highlighted the inhibitory effect of WA on NFκB signaling pathways in these cell types. WA suppressed *Helicobacter Pylori* induced NFkB activation and downstream signaling pathways in bone marrow derived dendritic cells and macrophages^48^. WA inhibited NFkB nuclear translocation in LPS induced bone marrow-derived macrophages^49^. In T and B lymphocytes, WA inhibited NFkB nuclear translocation, proliferation and secretion of Th1 and Th2 cytokines^26^. Thus, inhibitory effects of WA on DCs and T cells observed in the current study could be relayed through suppression of NFκB signaling pathways, warranting further investigations.

In conclusion, the present study demonstrated that WA inhibits allogeneic responses in mouse model of islet transplantation. WA suppresses DC maturation and several chemotactic and proinflammatory cytokines released by islets ex vivo. Our in vivo and in vitro observations emphasize the inhibitory effects of WA on both the innate and adaptive immune response. Islet graft survival in the peri-transplant period is central to achieving long-term graft function and insulin independence, as observed in our current study. We observed that anti-inflammatory actions of WA were conserved between mouse and human cell types including islets and immune cells, indicating that WA has clinically relevant applications in humans. As WA is a C28 steroidal lactone with established anti-tumor, anti-diabetic and anti-inflammatory actions, WA’s effects observed in our current study are not specific to inhibition of allogeneic rejection but yet another example of the therapeutic potential of WA. Further investigations are necessary to investigate the effects of combination therapy with existing low-dose systemic immunosuppressive regimen on allo-islet transplantation outcomes.

## Methods

Animal experimentation was approved by the Institutional Animal Care and Use Committee at Baylor Scott & White Research Institute and carried out in compliance with the ARRIVE guidelines. All methods were performed in accordance with the relevant guidelines and regulations.

### Diabetes induction, islet transplantation and WA treatment of recipient mice

Male BALB/c and C57BL/6N mice (aged 6–7 weeks) were purchased from Envigo (Houston, TX) and housed under specific pathogen-free conditions at Baylor Scott and White Research Institute. Diabetes was induced in C57BL/6N recipient mice by intravenous injection of streptozotocin (180 mg/kg body weight) (Sigma-Aldrich, St. Louis, MO). Mice with persistent nonfasting blood glucose levels > 400 mg/dL 2–3 days after streptozotocin injection were considered diabetic. BALB/c mouse islets (n = 300) pretreated with WA were transplanted into the portal vein of recipient mice. Recipients were treated with 0.25 mg/kg WA intraperitoneally 1 h before transplant and then treated for 7 days after transplant or until an allogeneic rejection occurred. To compare the immunosuppressive effect, recipient mice received an equal number of nontreated islets and intraperitoneal administration of low-dose FK506 (0.5 mg/kg) once a day from day 0 (1 h before transplant) until the event of allogeneic rejection. Nonfasting blood glucose levels and body weight were monitored 3 times a week in all recipients until 60 days after islet transplantation. Blood glucose was measured using a StatStrip Xpress2 Glucose Meter (Nova Biomedical, Waltham, MA). Normoglycemia after transplantation was defined as 2 consecutive blood glucose readings < 200 mg/dL. Graft rejection was defined as 2 consecutive hyperglycemias > 400 mg/dL. The first day of confirmed hyperglycemia was defined as the day of rejection. An autopsy of the liver was performed in recipients with functional grafts > 60 days after transplantation to ensure an engrafted allogeneic islet graft. The spleen of the recipients was also removed along with the liver and used for the analysis of regulatory T (Treg) cells. Mouse islet grafts with the liver were processed for immunohistochemical analysis as described previously^20^. Details on the animals, mouse islet isolation, and stains for immunohistochemistry studies are provided in the Supplementary Information.

### Mice lymphocyte proliferation assay

Splenocytes and lymph node cells from C57BL/6N and BALB/c mice were isolated using standard protocol. Spleens from 2–3 mice were pooled together and disintegrated through a 70 µm cell strainer. Similarly, mesenteric lymph nodes from mice were grinded through the cell strainer to obtain lymph node cells. Splenocytes were then used for the isolation of CD3^+^ T cells using magnetic beads from the mouse T-cell isolation kit (STEMCELL Technologies, Cambridge, MA). The cells were then stimulated with a 1:1 ratio of CD3/CD28 beads (Thermo Fisher Scientific, Waltham, MA) for activation of T cells (per recommended protocol) and cultured for 5 days with or without WA (0.5 μg/mL). Flow cytometry analysis was performed, and each experiment was performed in triplicate and repeated 3 times.

### Human T-cell proliferation assay

Human peripheral blood mononuclear cells (PBMCs) were isolated from buffy coat blood samples of healthy adult donors using standard density gradient centrifugation. Cells at the interface were collected and washed 3 times in cold phosphate-buffered saline containing 2% fetal bovine serum and 2 mM ethylenediaminetetraacetic acid, disodium salt. After culture and incubation, PBMCs were stimulated with human CD3/CD28 activator beads (Thermo Fisher Scientific) in the presence or absence of WA (0.25 and 0.5 μg/mL). Flow cytometry analysis was performed to assess T-cell proliferation after 5 days. Details on flow cytometry analysis and the culture of mouse and human cells for the assay are provided in the Supplementary Information.

### Additional studies on human PBMCs

Mixed lymphocyte reaction (MLR) was performed as described previously^50^. Human DC maturation assays were completed, with mature DCs generated as described previously^51^. In addition, the exosomal content of isletokines was analyzed. Details for all studies are provided in the Supplementary Information.

### Statistical analysis

Statistical analysis was done using GraphPad Prism 6.0 (GraphPad Software Inc, San Diego, CA) or SPSS Statistics Version 25 (IBM Corp, Armonk, NY). Comparisons between groups were determined by a two-tailed unpaired *t* test or one-way analysis of variance with post hoc test wherever appropriate. Survival data were analyzed using SPSS and shown by Kaplan–Meier methods. Islet graft survival between experimental groups was compared using the pairwise log-rank test. Flow cytometry data were analyzed using FlowJo software, version 10.7.2 (Tree Star Inc., Ashland, OR). All data are presented as mean ± standard error of the mean. *P* values < 0.05 were considered statistically significant.

## Supplementary Information

Supplementary Information.

## Data Availability

The data that support the findings of this study are available on request from the corresponding author.

## Abbreviations

AP-1: Activator protein 1
CFSE: Carboxyfluorescein succinimidyl ester
COX-2: Cyclooxygenase-2
DC: Dendritic cell
FACS: Fluorescence-activated cell sorting
GVHD: Graft-versus-host disease
HLA-DR: Human leukocyte antigen–DR isotype
IFN: Interferon
IKKβ: Inhibitor of nuclear factor kappa-B subunit beta
IL: Interleukin
iNOS: Inducible nitric oxide synthase
IP-10: Interferon-gamma-induced protein-10
MCP-1: Monocyte chemoattractant protein-1
MHC: Major histocompatibility complex
MLR: Mixed lymphocyte reaction
NFAT: Nuclear factor of activated T cells
NF-κB: Nuclear factor-kappa B
PBMC: Peripheral blood mononuclear cell
TCR: T-cell receptor
TNF-α: Tumor necrosis factor-alpha
Treg: Regulatory T cell
WA: Withaferin A
