# Supplementary material for: Withaferin A inhibits lymphocyte proliferation, dendritic cell maturation in vitro and prolongs islet allograft survival
Source: Sci Rep. 2021 May 21;11:10661. doi: 10.1038/s41598-021-90181-y (PMC8140140; doi:10.1038/s41598-021-90181-y)
Supplement: Supplementary file 1 — Supplementary Information. [file 41598_2021_90181_MOESM1_ESM.pdf]

Supplementary Methods Information

Withaferin A inhibits lymphocyte proliferation, dendritic cell maturation in vitro and prolongs islet allograft survival

Kenjiro Kumano, MD, PhD, Mazhar A. Kanak, PhD, Prathab Balaji Saravanan, PhD,
Jean Philippe Blanck, Yang Liu, MD, PhD, Srividya Vasu, PhD,
Michael Lawrence, PhD, Bashoo Naziruddin, PhD

Animals

Male BALB/c and C57BL/6N mice (aged 6-7 weeks) were purchased from Envigo (Houston, TX) and housed under specific pathogen-free conditions at Baylor Scott and White Research Institute. Diabetes was induced in recipient mice by intravenous injection of streptozotocin (180 mg/kg body weight) (Sigma-Aldrich, St. Louis, MO). Mice with persistent nonfasting blood glucose levels >400 mg/dL 2-3 days after streptozotocin injection were considered diabetic. Animal experimentation was approved by the Institutional Animal Care and Use Committee at Baylor Scott & White Research Institute.

Mouse islet isolation with WA treatment

Mouse pancreas perfusion using Collagenase Type V (1 mg/mL) (Sigma Aldrich) and islet isolation were performed as described previously.[1] Donor pancreases were perfused with collagenase solution supplemented with 0.5 µg/mL WA (Enzo Life Science, Farmingdale, NY). Isolated islets were cultured in Dulbecco's Modified Eagle's medium (Thermo Fisher Scientific, Waltham, MA) supplemented with 10% fetal bovine serum (Sigma-Aldrich) and 1% Antibiotic-Antimycotic (Thermo Fisher Scientific) at 24°C overnight before transplantation. Before islet transplantation, 300 hand-picked islets were pretreated with 0.5 µg/mL WA at 37°C for 30 minutes.

Immunohistochemistry

For insulin and glucagon staining, after antigen retrieval and blocking, fixed sections were stained with guinea pig anti-insulin (ABCAM, Cambridge, MA) and mouse IgG1 anti-glucagon (ABCAM), followed by incubation with secondary antibodies (Goat anti-Guinea Pig IgG (H+L) Highly Cross-Adsorbed Secondary Antibody, Alexa Fluor 568; Goat anti-Mouse IgG1 Cross-Adsorbed Secondary Antibody, Alexa Fluor 488; Thermo Fisher Scientific). Finally, sections were counterstained with 4′,6-diamidino-2-phenylindole. All slides were mounted with ProLong Gold Antifade Mountant (Thermo Fisher Scientific) and observed under the Nikon Ti-E inverted microscope with NIS Elements.

Cell proliferation assays

For the mouse cells, isolated T cells and lymph node cells were cultured in Roswell Park Memorial Institute (RPMI) 1640 medium (Thermo Fisher Scientific) supplemented with 20 U/mL human interleukin (IL)-2 (Sigma-Aldrich), 10% fetal bovine serum, 55 mM 2-mercaptoethanol (Thermo Fisher Scientific), 100 U/mL penicillin, and 100 mg/mL streptomycin (Thermo Fisher Scientific). Lymph node cells and T cells were labelled with carboxyfluorescein succinimidyl ester (CFSE) at 1 uM to assess proliferation.

Human PBMCs were cultured in RPMI 1640 medium containing 10% fetal bovine serum, penicillin/streptomycin, 55 mM 2-mercaptoethanol, and recombinant human IL-2 (20 U/mL) (PeproTech, Rocky Hill, NJ). Human PBMCs were incubated with CFSE according to the manufacturer's instructions (Invitrogen, Carlsbad, CA)..

Mixed lymphocyte reaction

Briefly, human PBMCs (responder cells) were incubated with CFSE according to the manufacturer's instructions (Invitrogen). Allogeneic human spleen cells (stimulator cells) were obtained from a patient undergoing splenectomy at our center with informed consent and pretreated with 50 mg/mL mitomycin C (Sigma-Aldrich) at 37°C for 30 minutes. The responder cells were cocultured with allogeneic stimulator cells at equal cell seeding densities (1×10^5 cells) in RPMI 1640 medium (Thermo Fisher Scientific) supplemented with 20 U/mL human IL-2 (Sigma-Aldrich), 10% fetal bovine serum, 55 mM 2-mercaptoethanol (Thermo Fisher Scientific), 100 U/mL penicillin, and 100 mg/mL streptomycin (Thermo Fisher Scientific) in 96-well U-bottomed plates at 37°C in a humidified atmosphere with 5% carbon dioxide. The cells and media were collected after 1 and 6 days of incubation for proliferation analysis and cytokine secretion assessment, respectively. The anti-human CD4 antibody was used to determine $CD4^+$ T cells, and then the CD4-gated cell population was assessed by flow cytometry analysis to detect changes in CFSE fluorescence. 7-Aminoactinomycin D (7-AAD)–positive cells were excluded as dead cells. For the WA treatment group, the cells were cultured in the same medium containing WA 0.25 to 0.50 μg/mL using the same protocol. The concentration of human IL-2 or interferon (IFN)-γ produced in the culture supernatants was determined by enzyme-linked immunosorbent assay kits (Biolegend, San Diego, CA).

For T-cell subset analysis, human PBMCs were cocultured with irradiated allogeneic human splenocytes in the RPMI 1640 medium containing WA 0.50 μg/mL (Cayman Chemicals, Ann Arbor, MI) for 7 days. $CD8^+IFN\text{-}\gamma^+$ T cells, $CD4^+IL\text{-}4^+$ T cells, and $CD4^+CD25^+Foxp3^+$ T cells (Treg) were analyzed by flow cytometry analysis. All antibodies were purchased from eBioscience. Fold change was calculated by dividing each T-cell subset increase (%) by the mean value of the T-cell subset increase of the control group (PBMCs only) (%).

Human DC maturation assay

Mature DCs were generated as described previously.[2] Briefly, immature DCs were generated from whole PBMCs (2.5×10^6 cells/mL) by cultivation in RPMI 1640 medium supplemented with 10% fetal bovine serum (Sigma-Aldrich) and 1X Antibiotic-Antimycotic (Thermo Fisher Scientific) containing granulocyte-macrophage colony-stimulating factor (20 ng/mL) (PeproTech), IL-4 (40 ng/mL) (PeproTech), and IFN-β (1000 U/mL) (PeproTech) in 24-well plates at 37°C and 5% carbon dioxide for 1 day. These immature DCs were matured by incubation in the presence of 10 ng/mL of TNF-α (PeproTech), 10 ng/mL of IL-1β (PeproTech), and 1 μg/mL of prostaglandin E2 (PeproTech) for 1 day. Maturation was assessed by the following phenotype: $CD14^{low}$, $CD86^{high}$, and $CD83^{high}$, a typical marker of matured myeloid DCs.[3] For the WA treatment group, PBMCs were cultured in the DC-maturing medium containing WA 0.15-0.25 μg/mL using the same DC maturation protocol.

Exosome analyses

For analysis of exosomal content of isletokines, human islets (Integrated Islet Distribution Program, City of Hope, CA) were incubated in clinical-grade CMRL-1066 media (Corning Mediatech, Manassas, VA) supplemented with 10% human albumin at 37°C. Purified human islets of about 2500 islet equivalents (n = 3) were exposed to WA (1.0 μg/mL) alone for 24 hours or to a cytokine cocktail of IL-1β (100 U/mL) + IFN-γ (1000 U/mL) + TNF-α (1000

U/mL) for 24 hours with or without 3 hours of WA (1.0 μg/mL) pretreatment. After the treatment, the exosomes released from islet culture were isolated by the polyethylene glycol–based overnight precipitation method and enriched with ultrafiltration. Total exosome protein was quantified by micro BCA protein assay (Thermo Fisher Scientific). Exosomes were characterized by transmission electron microscopy, nano-sight tracking analysis, and Western blotting for Flotillin-1 and CD-9 markers. The total quantity of isletokines present in the released exosomes was quantified by multiplex Luminex assay using Milliplex human cytokine/chemokine magnetic bead 5-plex panel (Merck-Millipore, Burlington, MA) according to the manufacturer's instructions. All measurements were performed on a Magpix Luminex instrument, using xPonent 4.2 (Luminex, Austin, TX) and Bio-Plex Manager 6.1 (Bio-Rad, Hercules, CA) software. For analysis of macrophage activation by islet exosomes, THP cells were treated with islet exosomes, and then total RNA was isolated using TRIzol and column-based Direct-zol RNA isolation kit (ZYMO Research, Irvine, CA) and converted to complementary DNA using a Universal cDNA synthesis kit (Thermo Fisher Scientific) following the manufacturer’s instructions. Real-time polymerase chain reaction (PCR) was performed using the Stratagene Mx3000P system with RT2 SYBR Green qPCR mastermix (SABiosciences, Frederick, MD). Thermal profile and cycling were performed at 95°C for 10 minutes for cycle 1 followed by 45 cycles of 95°C for 10 seconds and 60°C for 30 seconds. RT2 qPCR primer assays (SABiosciences) were used to find the relative mRNA expression for inducible nitric oxide synthase (iNOS) and cyclooxygenase-2 (COX-2) in these cells.

Flow cytometry analysis

Fluorescence-activated cell sorting (FACS) for Treg analysis was performed using eBioscience^{TM} Mouse Regulatory T Cell Staining Kit #1 (Thermo Fisher Scientific). The proliferation of mouse and human lymphocytes was measured by flow cytometry analysis of CFSE with the decay of CFSE reflecting T-cell proliferation. For human cell experiments, the fluorescent dye-labeled monoclonal antibodies against human cell surface molecules included anti-CD3, anti-CD8, anti-CD14, anti-CD19, anti-CD83, anti-CD86, 7-AAD (BD Biosciences, San Jose, CA), anti-CD4 (eBioscience, San Diego, CA), anti-CD11c (Biolegend), and anti–human leukocyte antigen–DR isotype (HLA-DR) (Invitrogen). FcR Blocking Reagent (Miltenyi Biotec Inc., Auburn, CA) was used to block the binding of antibodies to the Fc receptor of human Fc receptor-expressing cells. Fluorescence minus one controls for each antibody were utilized to establish gates correctly and to determine the frequency of positively stained cells. The cells were analyzed by a BD Cant II (BD Biosciences) and FlowJo software (Tree Star Inc., Ashland, OR). Proliferation rate was computed using proliferation modeling tool in FlowJo software.

References

1. Kumano K, et al. Grafting Islets to a Dissected Peritoneal Pouch to Improve Transplant Survival and Function. *Transplantation.* **104**, 2307-2316 (2020).
2. Kodama A, Tanaka R, Saito M, Ansari AA, Tanaka Y. A novel and simple method for generation of human dendritic cells from unfractionated peripheral blood mononuclear cells within 2 days: its application for induction of HIV-1-reactive CD4(+) T cells in the hu-PBL SCID mice. *Front Microbiol.* **4**, 292 (2013).
3. Ohshima Y, et al. Expression and function of OX40 ligand on human dendritic cells. *J Immunol.* **159**, 3838-3848 (1997).

Supplementary Figure S1

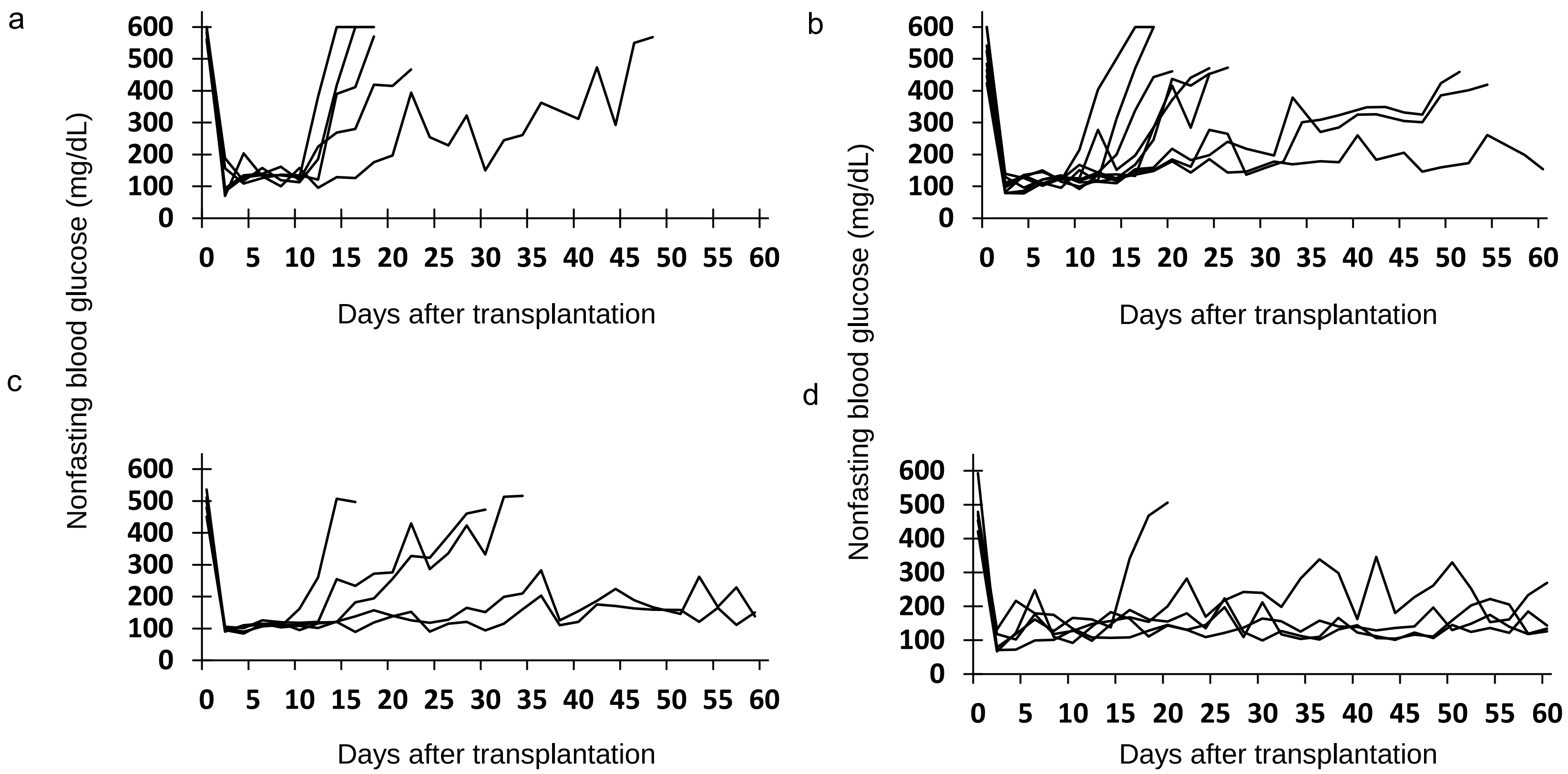

Supplementary Figure S1. Individual blood glucose readings for a cohort of C57BL/6N recipient mice.
Recipient mice were assigned to four groups; a) Control (no treatment) (n=5), b) 1.25 mg/kg WA 7-day treatment (n = 9), c) low-dose FK506 daily treatment (n=5), d) 1.25 mg/kg WA daily treatment (n=5).

Supplementary Figure S2

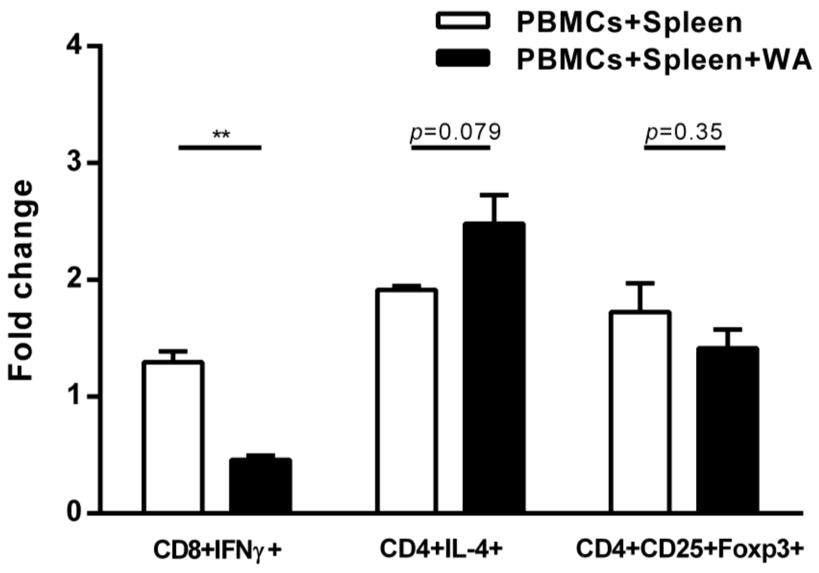

Supplementary Figure S2. T-cell subset analysis using mixed lymphocyte reaction. Human PBMCs were cocultured with irradiated allogeneic human splenocytes in a medium containing WA 0.50 μg/mL for 7 days. Cell differentiation was analyzed by FACS. The differentiation ratio (fold change) was calculated by dividing each T-cell subset increase (%) by the mean value of T-cell subset increase of the control group (PBMCs only, data not shown) (%). The experiment was repeated 3 times. ** $P < 0.01$.

Supplementary Figure S3:

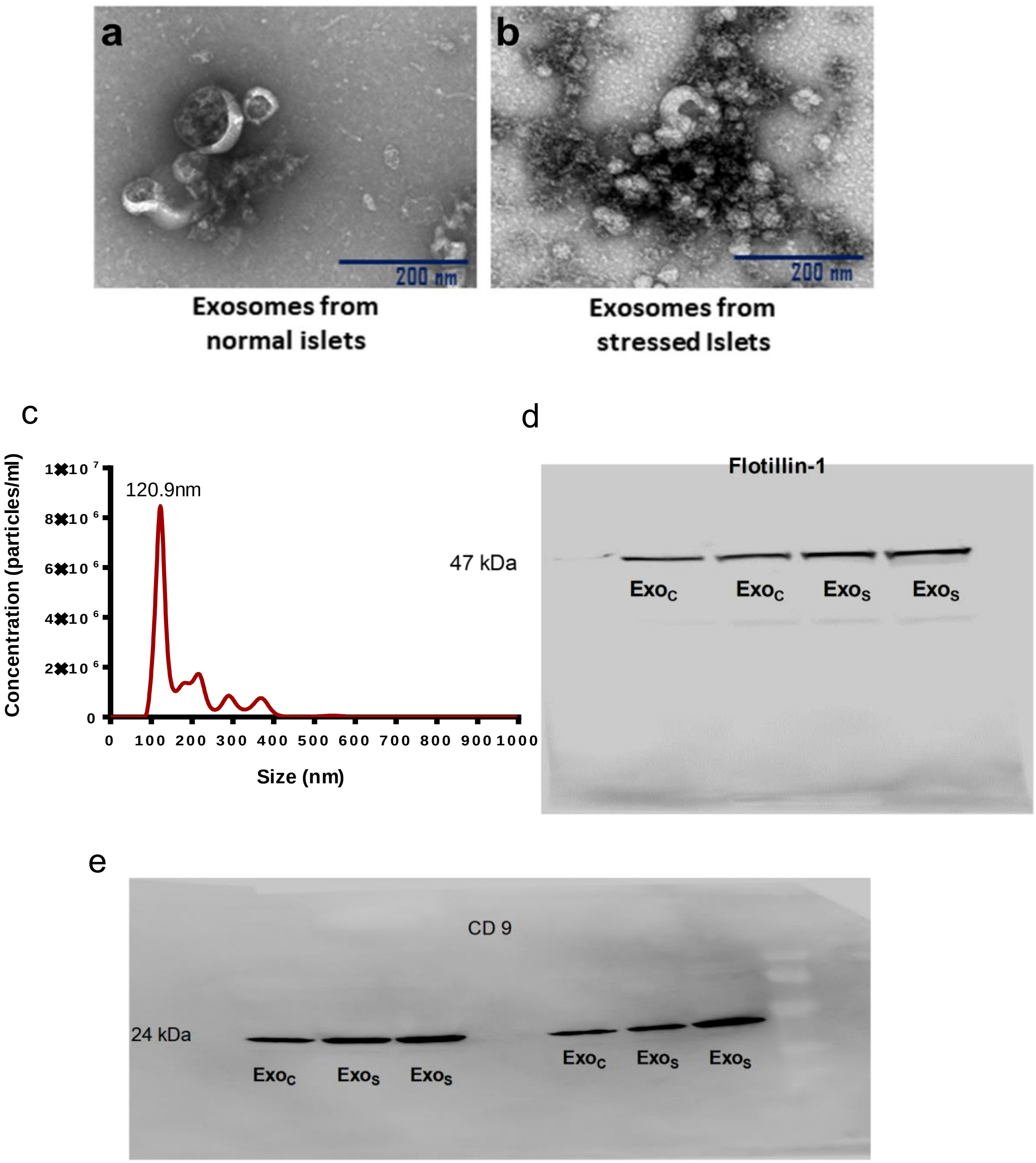

Supplementary Figure S3. Characterization of exosomes. Exosomes isolated from islet culture supernatant were characterized by **(a, b)** morphological visualization using transmission electron microscopy, **(c)** size determination by nanoparticle tracking analysis, and **(d, e)** immunoblotting for exosomal markers Flotillin-1 and CD-9. Exo$_C$, exosomes from control; Exo$_S$, stressed islet culture media.
